# Cooperative Relaxation Governs the Viscosity of Ionic Liquids

**DOI:** 10.1021/acs.jpclett.6c02640

**Published:** 2026-10-01

**Authors:** Shurui Miao, Bela Farago, Ingo Hoffmann, Rob Atkin, Michael Gradzielski, Gregory G. Warr

**Affiliations:** † School of Chemistry and University of Sydney Nano Institute, The University of Sydney, Sydney, NSW 2006, Australia; ‡ Physical and Theoretical Chemistry Laboratory, 6396 University of Oxford, Oxford OX13QZ, United Kingdom; § Institut Max von Laue-Paul Langevin (ILL), 71 avenue des Martyrs, CS 20156, Cedex 9, F-38042, Grenoble, France; ∥ School of Molecular Sciences, The University of Western Australia, Perth, WA 6009, Australia; ⊥ Stranski-Laboratorium für Physikalische und Theoretische Chemie, Institut für Chemie, Technische Universität Berlin, Straße des 17. Juni 124, Sekr. TC7, D-10623, Berlin, Germany

## Abstract

Ionic liquids are promising solvents for batteries, biomass processing and critical mineral recovery, but their viscosities can exceed those of conventional molecular solvents by several orders of magnitude, impeding mass and charge transport and complicating process design. Using complementary neutron spin echo spectroscopy and nuclear magnetic resonance diffusometry, we probe local and long-range dynamics of three protic ionic liquids over time scales from 10^–12^ to 10^–3^ seconds. We identify a fast relaxation with closely corresponding self and collective time scales, indicating cooperative rearrangement of the surrounding molecular network. Its characteristic time scale correlates with bulk viscosity, while its temperature dependence reveals distinct Arrhenius activation energies depending on whether extended networks are dominated by hydrogen bonding or electrostatic interactions. These results link cooperative network relaxation to macroscopic viscosity and show how molecular structure can tune the energetic cost of structural relaxation through network connectivity.

## Graphical abstract

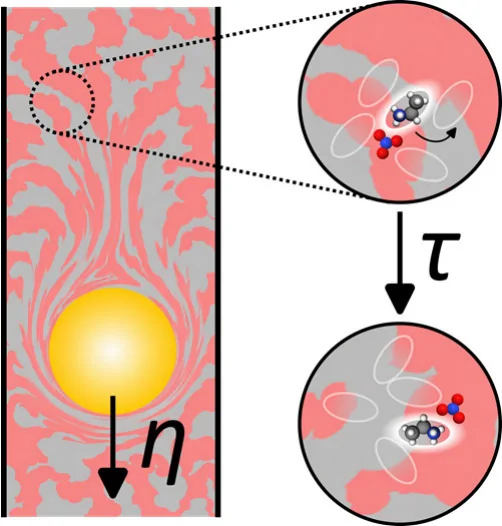

Ionic liquids (ILs) are salts that melt at or near room temperature. They are widely explored as designer solvents for applications in energy storage,^1,2^ separations,^3^ light-emitting cells,^4^ and biomass processing,^5^ due to the ability to tune intermolecular interactions, nanostructure, and transport properties through cation and anion design. However, ILs are typically at least an order of magnitude more viscous than conventional molecular solvents, with viscosities that are also strongly dependent on temperature and impurities. This high and variable viscosity impedes ionic transport in electrochemical applications and limits pumping, mixing and mass transfer in industrial separations and processing, complicating process design and increasing operational costs. Despite extensive efforts to relate viscosity to ion size, flexibility, and intermolecular interactions, the molecular relaxation processes underlying their high viscosity remain unresolved.^6–12^ This lack of understanding of how molecular features translate into macroscopic viscosity limits the rational design of IL-based functional electrolytes with optimized transport properties.

Empirical descriptions of liquid viscosity based on free volume, molecular size and pairwise interactions are often insufficient for ionic liquids, where long-range Coulomb interactions and specific directional forces, such as hydrogen bonding, generate strongly correlated dynamics.^13–15^ In addition, many ILs exhibit nanoscale segregation into polar and apolar domains, forming *amphiphilic nanostructures*.^16–18^ Such nanoscale segregation also creates spatially distinct thermodynamic environments, such that the local chemical potential and solvation environment experienced by an ion can depend on its position within the heterogeneous liquid.^19,20^ Consequently, ion motion through these domains reflects not only molecular interactions but also the free-energy landscape associated with the local environment. This static structural heterogeneity can therefore contribute to complex transport behaviour, such as the diffusion-viscosity decoupling.^21,22^

Identifying the molecular relaxation processes underlying viscosity is particularly challenging in these nanostructured ILs, where multiple anisotropic intermolecular forces govern the continuous reorganisation of local structures.^23–25^ Broadly, transport and relaxation in ILs have been described through many physical frameworks, including the hole theory and mode-coupling descriptions of correlated dynamics.^25–27^ These descriptions capture different aspects of transport across different length and time scales, but the underlying molecular motions are not clear. Extensive computational studies have suggested that cooperative, sub-diffusive motions associated with ion-network reorganisation may provide a mechanistic link between nanoscale structural heterogeneity and macroscopic viscosity.^28–32^ Experimentally connecting these processes remains challenging because techniques such as rheology, dielectric and neutron spectroscopy probe different correlation functions over different spatial and temporal windows. Here we have combined the recent development in wide-angle neutron spin echo spectroscopy (NSE) with pulsed-field-gradient nuclear magnetic resonance (PFG-NMR) to connect long-range self-diffusion with local self and collective relaxation.

NSE is one of few experimental techniques that can probe the spatial and temporal regimes relevant to local dynamics in liquids.^33–36^ Previous NSE studies of ILs and concentrated electrolytes were either limited in accessible spatiotemporal range or did not explicitly account for amphiphilic nanostructure.^37–40^ Here, wide-angle NSE provides access to dynamics across a broader *q*-range, while isotopic contrast allows self and collective contributions to be distinguished. We investigate three protic ionic liquids whose nanostructures span a well-defined progression in network connectivity from a three-dimensional hydrogen-bond network to increasingly segregated morphologies (Figure 1).^41–43^ We show that cooperative network relaxation is closely linked to their bulk viscosity. To resolve this process from other modes of motion, we combine wide-angle NSE (4 × 10^–10^ – 5 × 10^–9^ m, 5 × 10^–12^ – 1 × 10^–8^ s) with PFG-NMR measurements of long-range self-diffusion (10^–6^ m, 10^–3^ s). This experimental framework enables local relaxation dynamics to be interpreted with diffusive transport.

**Figure 1:**
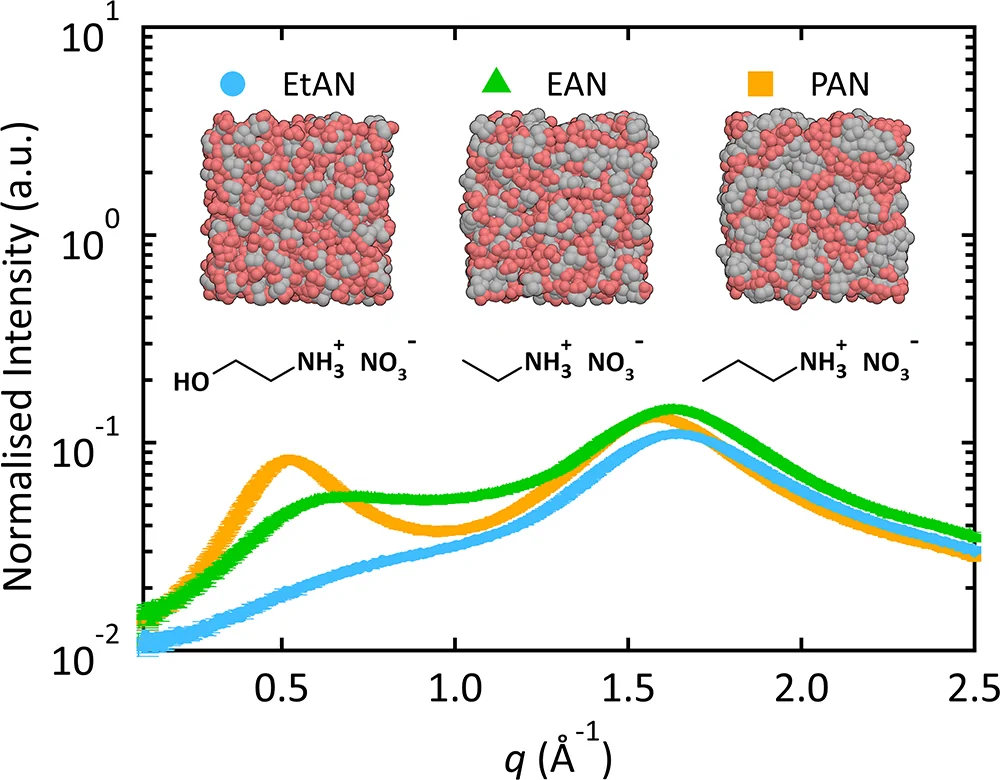
Systematic development of nanostructure in model ionic liquids. X-ray scattering patterns of ethanolammonium nitrate (EtAN, blue), ethylammonium nitrate (EAN, green) and propylammonium nitrate (PAN, yellow), together with liquid models derived from neutron diffraction. The scattering patterns reflect the increasing development of nanostructure from a shoulder near 0.76 Å^–1^ in EtAN, into a distinct peak in EAN at 0.63 Å^–1^, and PAN at 0.53 Å^–1^, corresponding to increasing correlation lengths of 8.3, 10, and 12 Å, respectively. Color-coded polar (red) and apolar/alkyl (grey) networks show a cation-controlled amphiphilic nanostructure replotted from published studies.^41–43^

NSE spectra of hydrogen-containing samples primarily reveal self-correlated relaxations such as diffusion, since scattering from ^1^H is predominantly incoherent in nature due to its large, spin-incoherent scattering cross-section.^44^ Neutron polarization analysis confirms that coherent scattering fractions are below 0.12 at all accessible wavevectors, *q* (in reciprocal space), for all three ILs (see Figure S5). Therefore, the measured intermediate scattering functions, *I*(*q*,*t*)/*I*(*q*,0), were initially fitted to a single exponential term according to Fick’s law (details in SI). Figure 2A shows that the effective self-diffusion coefficients (*D*_Effective_) derived from such fits agree quantitatively with bulk diffusion coefficients measured by PFG-NMR at large length scales (*q* < 0.6 Å^–1^). At shorter length scales, *D*_Effective_ clearly deviates from their bulk values, signalling that the Fickian model is becoming insufficient.

**Figure 2:**
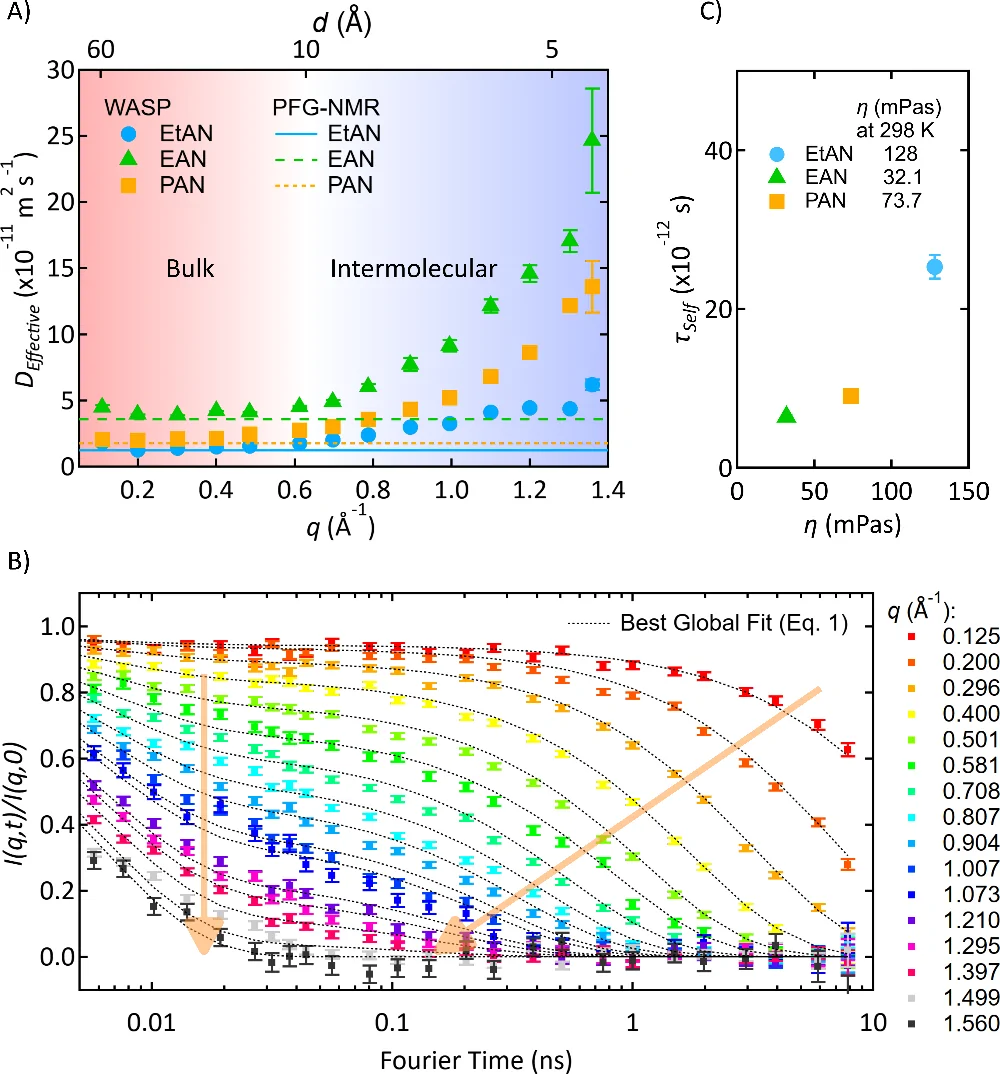
Experimental neutron spin echo data and fits. (A) Effective diffusion coefficients (*D*_Effective_) from NSE by fitting a diffusion-only model (details in SI), showing agreement with experimental PFG-NMR (lines) in the low-*q* regime. Real space distances, *d* = 2π/*q*, are shown as the top axis. (B) Representative NSE intermediate scattering functions and fits (eq 1). Two modes of relaxation are clearly visible, from left to right, the two arrows correspond to (i) the approximately *q*-independent fast mode; and (ii) the *q*-dependent Fickian diffusion. (C) Correlation between IL bulk viscosity and fitted τ_Self_ relaxation time using eq 1, uncertainty is smaller than the marker for EAN and PAN. Viscosity of ILs at 298 K are taken from literature.^47^

The bulk self-diffusion coefficients increase in order EtAN < PAN < EAN. This trend reflects differences in local intermolecular interactions and steric effects. In EtAN, the presence of a hydroxyl group allows a more extensive hydrogen bond network to form. On average, 4.45 hydrogen bonds form around cations in EtAN, whereas only 2.83 and 2.91 are present for cations in EAN and PAN, respectively.^41–43^ The slower diffusion of PAN relative to EAN may reflect its larger molecular size and molar volume,^45,46^ although the strongly interacting and nanostructured nature of these liquids precludes a simple continuum description of this effect.^21,22^

At intermolecular and shorter length scales (*q* > 0.6 Å^–1^), intermediate scattering functions reveal the emergence of an additional, faster, self-correlated relaxation process. The shapes of measured *I*(*q*,*t*)/*I*(*q*,0) shown in Figure 2B also suggests that more than one exponential decay is present (see Figures S2 and S7 for full datasets), highlighting that diffusion alone cannot account for the observed dynamics. Therefore, two dynamic processes occur in the accessible length- and time-scale window: a slower component characteristic of Fickian diffusion, and a faster relaxation that becomes increasingly prominent at higher *q*. This additional mode may be the missing, nondiffusive relaxation process required to account for viscosity-diffusion decoupling. To quantify this behaviour, we employ a biexponential model (eq 1, model choice discussed in the SI) to extract the characteristic relaxation time of the fast mode as τ_Self_.

1 $${I\left(q,t\right)/I\left(q,0\right)}_{\mathrm{Inc}}=K\left(q\right)\times e^{-D_{\mathrm{NMR}}q^{2}t}\times \left(A\left(q\right)+\left(1-A\left(q\right)\right)\times e^{-t/\tau_{\mathrm{Self}}}\right)$$

where the prefactor *K*(*q*) accounts for intramolecular dynamics, such as vibrations and rotations, that occur on femtosecond time scales and therefore lie outside the experimental window.^48^ *A*(*q*) represents the relative contribution of diffusion to the total relaxation, with *A*(*q*) = 1 corresponding to purely Fickian diffusion. The self-diffusion coefficient, *D*_NMR_, is fixed to the value independently determined by PFG-NMR. *K*(*q*) and *A*(*q*) are fitted independently at each *q* (Figure S3). A single relaxation time, τ_Self_, representing the characteristic time scale of the fast self-correlated relaxation, is fitted globally across the entire *q*-range. This is consistent with its approximately *q*-independent behaviour over the region where the fast contribution is significant (Figures S3 and S4).

The fitted *K*(*q*) is close to 1 across the accessible *q*-range, indicating that contributions from relaxations faster than 5 ps are generally small, although some attenuation is observed at high *q* (see Figure S3).^48,49^ These unresolved fast motions are therefore primarily absorbed into *K*(*q*), but contributions from local intramolecular dynamics at the highest *q* cannot be completely excluded. *A*(*q*) decreases from 1 to 0 with increasing *q*, reflecting the growing contribution of the fast relaxation mode at shorter length scales. This behaviour indicates that the fast dynamics arise from spatially confined motions and it becomes the dominant relaxation process at *q* > 0.8 Å^–1^, corresponding to length scales smaller than the domain size in these nanostructured liquids.^41–43^ We emphasise that the static nanostructure identified by scattering does not itself establish dynamic heterogeneity; rather, the present NSE measurements independently reveal the spatial dependence of the relaxation dynamics. Interestingly, τ_Self_ varies consistently with bulk viscosity across the three ILs (Figure 2C), suggesting a connection between this fast relaxation and macroscopic viscosity that motivates further analysis.

To investigate how this self-relaxation is coupled to its local environment, we studied collective relaxation processes. The relative contributions of the collective (coherent) versus self-correlated (incoherent) signals in neutron scattering can be enhanced through isotopic substitution of hydrogen with deuterium. Building on our understanding of the hydrogenous systems, we performed an incoherent correction to deuterated datasets to further enhance the contribution from collective dynamics (details in SI). This removes the self-contribution arising from the spin incoherence of hydrogen, but it does not account for the residual effect of isotope incoherence that arises whenever multiple isotopes of any element are present. Therefore, although the corrected *I*(*q*,*t*)/*I*(*q*,0) is dominated by coherent signals, a small residual diffusion contribution due to isotope incoherence is also present. As a result, we fitted the corrected *I*(*q*,*t*)/*I*(*q*,0) using the same biexponential function as in the incoherent analysis (analogous to eq 1, see eq S5). The resulting fit yields a time constant, τ_Coll_, that predominantly reflects collective dynamics.

We found that within each IL, the fitted self (τ_Self_) and collective (τ_Coll_) relaxation times are similar (Figure 3A). This suggests that local, individual molecular motions are accompanied by cooperative rearrangements of the surrounding network, giving rise to correlated self and collective dynamics. This is expected in these heterogeneous liquids, where molecular motion changes the local solvation and thermodynamic environment experienced by an ion, requiring correlated reorganisation of its surroundings to maintain a favorable local environment. Such coupling should be particularly prevalent in liquids containing extended interaction networks, whether sustained by hydrogen bonding, electrostatic interactions, or both, as in ILs. This coupling between self and collective relaxation also bears a resemblance to the structural α-relaxation characteristic of glass-forming liquids.^50^ In supercooled liquids, α-relaxation reflects cooperative escape from transient molecular cages, and its characteristic time scale is closely linked to macroscopic viscosity.^51^ The correlation of τ_Self_ with viscosity, together with the correspondence between τ_Self_ and τ_Coll_, therefore suggests that the relaxation observed here represent the microscopic counterpart of an α-like structural relaxation in these ILs. Nevertheless, while many ILs readily supercool and exhibit glass-forming behaviour,^52–54^ the present measurements are performed above the glass-transition regime and therefore do not establish a direct identification of τ_Coll_ with the conventional α-relaxation time. Figure 3B schematically illustrates this cooperative mechanism, where the motion of a single cation (τ_Self_) is accompanied by reorganisation of the surrounding charge and apolar networks (τ_Coll_), represented by the evolution of the red and grey regions. Similar coupling between self and collective dynamics has also been reported in water, where molecular rotation is coupled to the collective reorganisation of the hydrogen-bond network.^48,55,56^ This rearrangements have been identified as key determinant of viscous behaviour in aqueous electrolytes.^56^

**Figure 3:**
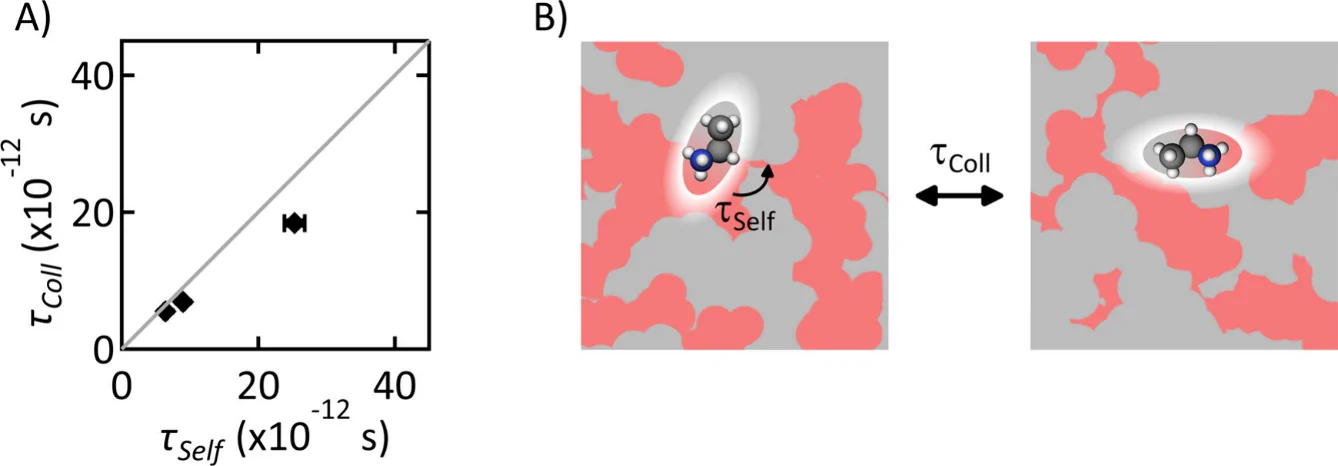
Correlated self and collective dynamics. (A) Correlation between fast self and correlated relaxation times, τ_Self_ and τ_Coll_, relaxation times for three ILs at 298 K, errors are smaller than the marker are not shown. The grey diagonal line shows perfect positive correlation to guide the eye. (B) Schematic shows how motion by one molecule (self) correlates to local rearrangement (collective) under constraints such as charge neutrality, density conservation, and directional intermolecular interactions.

The correspondence among the bulk viscosity, the self, and collective relaxation times, suggests a connection between collective relaxation and the stress relaxation underlying viscosity. We therefore examine the temperature dependence of τ_Coll_ to probe the energetic dependence of this relaxation process. Figure 4 reveals a clear distinction between the temperature dependence of bulk self-diffusion and cooperative network relaxation. The activation energy for self-diffusion follows the trend EtAN > PAN > EAN, due to differences in local intermolecular interactions and steric constraints. In contrast, the effective activation energies associated with cooperative relaxation clearly distinguish EtAN from the other two ILs. EtAN displays a substantially larger barrier, whereas in EAN and PAN this barrier is much smaller. These results indicate that although all three ILs contain similar classes of intermolecular interactions, the energetic cost of cooperative network reorganisation is not determined solely by the strength of local interactions.

**Figure 4:**
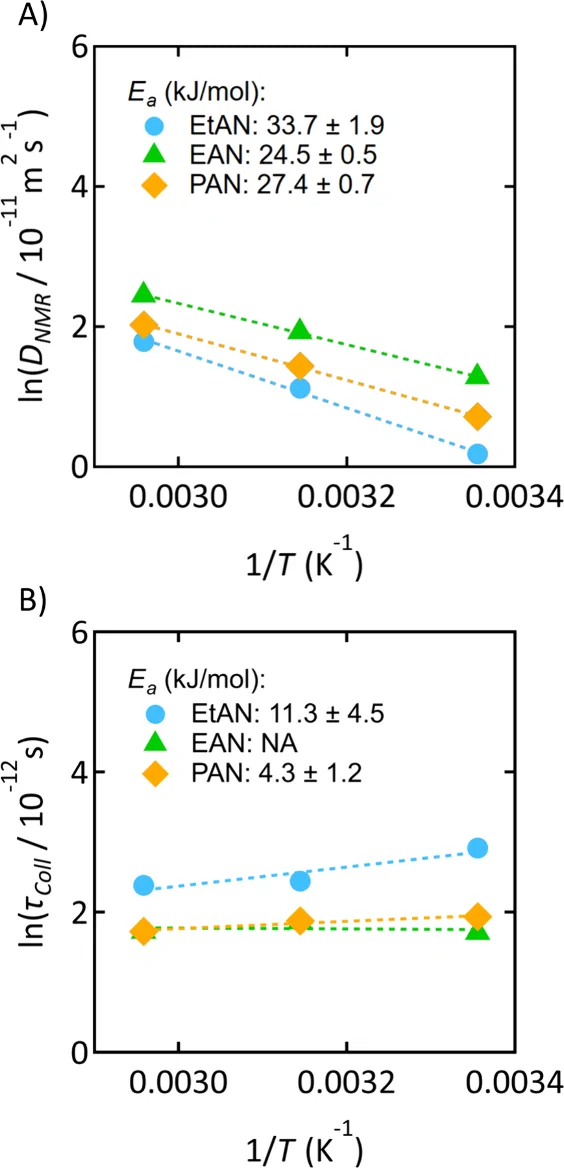
Activation energy of diffusion and fast cooperative dynamics. Temperature dependence of (A) self-diffusion coefficients (*D*_NMR_, from PFG-NMR) and (B) cooperative dynamics (τ_Coll_, from NSE). Data for each IL is fitted to the Arrhenius equation to extract an effective activation energy (*E*_a_). *E*_a_ from *D*_NMR_ is comparable for all three ILs, yet the *E*_a_ from τ_Coll_ shows qualitative difference between EtAN and other two ILs. Errors are smaller than the size of markers.

This distinction can be understood in terms of network connectivity. While both Coulombic interaction and hydrogen bonding contribute to the cohesive energy density in all three ILs, their network morphology differs due to amphiphilic self-assembly. Structural studies have shown a much more extensive hydrogen bonding network in EtAN than the other two ILs.^41–43^ Owing to its hydroxyl functional group and the lack of strong polar-apolar segregation, EtAN can form a dense, three-dimensional hydrogen bond network like water. In contrast, the amphiphilic nanostructure in EAN and PAN constrains their hydrogen bond networks to lower-dimensional motifs within the polar domains. This renders the hydrogen bond network in EAN and PAN more like one- or two-dimensional local structure with a high proportion of weaker and bent hydrogen bonds.^57,58^ Therefore, our results indicate that the hydrogen bond networks play a more significant role in regulating long-range structural relaxations in EtAN, whereas the isotropic long-range Coulombic interactions dominate in EAN and PAN.

We have identified cooperative network relaxation as a molecular process closely linked to macroscopic viscosity, providing mechanistic insight into how bulk transport properties can emerge from collective network relaxation in ILs and be tuned through molecular features. Across all three model ILs, we showed that subtle changes in molecular structure are sufficient to switch between Coulombic- and hydrogen-bond-dominated networks while preserving the same cooperative relaxation mechanism. This suggests that hydrogen bonding and electrostatic interactions can play equivalent roles at the level of cooperative relaxation among studied ILs, insofar as either can sustain an extended network whose reorganisation is linked to bulk viscosity. While these interactions compete in determining local structure, our results suggest that the energetic cost of cooperative rearrangement is governed primarily by the connectivity of the resulting network, rather than the energetic hierarchy of individual intermolecular interactions.

The coupling between self and cooperative relaxation, and their connection to viscosity, is reminiscent of the structural relaxation characteristic of glass-forming liquids, where increasingly cooperative molecular rearrangements govern viscous slowing. Together with existing studies of water and other neutral molecular liquids with strongly anisotropic intermolecular interactions, this similarity suggests that cooperative network relaxation may provide a common framework for understanding viscous dissipation beyond the model systems studied here.^48,49,55,56^ Nevertheless, whether cooperative relaxation represents a universal mechanism for viscous dissipation in disordered condensed matter remains to be established. Further investigation of simpler model liquids, such as argon and methane, where directional intermolecular interactions are absent and the interaction potentials are well characterized, would provide a direct test of this hypothesis.

## Supplementary Material



## Notes

Experimental details, materials and methods; Model fitting procedures; Complete fitted datasets and fitted parameters (PDF)

## #

Physical and Theoretical Chemistry Laboratory, University of Oxford, Oxford OX13QZ, United Kingdom
