# Supplementary material for: Cooperative Relaxation Governs the Viscosity of Ionic Liquids
Source: J Phys Chem Lett. 2026 Oct 1;17(41):11778–83. doi: 10.1021/acs.jpclett.6c02640 (PMC13679084; doi:10.1021/acs.jpclett.6c02640)
Supplement: Supplementary file 1 [file jz6c02640_si_001.pdf]

Supplementary Materials for

Cooperative relaxation governs the viscosity of ionic liquids

Shurui Miao[1,2], Bela Farago[3], Ingo Hoffmann[3], Rob Atkin[4], Michael Gradzielski[5], Gregory G. Warr[1*]*

[1]School of Chemistry and University of Sydney Nano Institute, The University of Sydney, NSW 2006, Australia

[2]Physical and Theoretical Chemistry Laboratory, University of Oxford, OX13QZ, United Kingdom

[3]Institut Max von Laue-Paul Langevin (ILL), 71 avenue des Martyrs, CS 20156, Cedex 9, F-38042 Grenoble, France

[4]School of Molecular Sciences, The University of Western Australia, WA 6009, Australia

[5]Stranski-Laboratorium für Physikalische und Theoretische Chemie, Institut für Chemie, Straße des 17. Juni 124, Sekr. TC7, Technische Universität Berlin, D-10623 Berlin, Germany

Corresponding author emails: shurui.miao@chem.ox.ac.uk; gregory.warr@sydney.edu.au

This PDF file includes:

Experimental Methods

Supplementary Text

Figs. S1 to S7

Tables S1 to S4

References (1 to 9)

Experimental Methods

Sample preparation

Ionic liquids (ILs) were produced by neutralising nitric acid (70%, Ajax) with corresponding alkyl amine (ethylamine, 66-72%, Sigma; propylamine, 99%, Sigma; ethanolamine 99%, Merck). All chemicals were used as received except ethanolamine. Ethanolamine was purified via vacuum distillation. The first 10 wt% of the distillation product was discarded. Subsequent ~ 80 wt% is collected as a colourless liquid, and purity was confirmed via 400 MHz ^{1}H NMR (Bruker). All reagents were diluted by Milli-Q water to ~20 wt%. Neutralisation was performed over an ice bath with vigorous stirring. The reaction temperature was maintained below 10 °C and the pH of the mixture was monitored using a Metrohm pH meter. Titration was stopped after reaching the endpoint with slightly more amine (pH ~ 6). The resultant solution was gently dried on a rotary evaporator at 40 ˚ C and 50 mbar for 3 hours. Further drying was performed by a high vacuum over > 48 hrs (0.2 mbar, room temperature). The final water content is < 0.2 wt% (Karl Fischer titration, Hanna Instrument). NMR was used to confirm stoichiometry and purity. Partially deuterated samples were prepared from hydrogenous samples by exchange with D_2O. IL and D_2O were mixed at 1:5 mole ratio and stirred for 10 min. Water was removed by rotary evaporator, and the exchange was repeated 5 times. Further drying was performed as described above, exchange rate confirmed to be > 99 mol% by NMR. Fully deuterated ILs were prepared following identical steps as the hydrogenous ILs. D_5-ethylamine (99%, Cambridge Isotope), D_4-ethanolamine (98%, Cambridge Isotope), and D_7-propylamine (98%, CDN Isotope) were used as received. Full deuteration is achieved post IL synthesis by exchange with D_2O as outlined above. Exchange rate confirmed to be > 99 mol% by NMR.

Small and wide-angle x-ray scattering

Scattering curves were obtained from a point-collimated Anton Parr SAXSpoint instrument. Liquid samples were loaded to 1 mm quartz capillaries and scattering patterns were accumulated on a Dectris Eiger R 1M detector. Scattering intensity was radially averaged and normalised to direct beam intensity. All measurements were obtained at 298 K over 10 minutes.

Pulsed-field gradient nuclear magnetic resonance (PFG-NMR)

Spectra of hydrogenous ILs were measured using a 400 MHz Bruker spectrometer and the built-in DOSY 2D program. Spectra were obtained at 298, 318, and 338 K without solvent. 16 linear steps between 5 and 95% of field strength are used, at each step, 8 scans were obtained to optimise signal-to-noise ratio, 2-8 s between each scan (depending on the sample, relaxation time is experimentally determined and multiplied by 5 to ensure all protons have sufficient time to relax between each scan). Obtained 2D data is reduced to 1D by using the peak height as the signal intensity. Self-diffusion coefficient is obtained by fitting to the Stejskal-Tanner equation.

High intensity wide-angle neutron spin echo (NSE)

NSE spectra were collected on the WASP beamline at the ILL for 40-60 seconds per detector image, about 100 images per spectrum. The three detector banks were covering the 5-100 degree scattering angles. 1 mL of samples were loaded into hollow cylindrical aluminium holders (0.1 mm gap thickness) in a glove box to minimise exposure to moisture. Measurements were performed at 298, 318, and 338 K using neutrons with a wavelength of 6 and 7 Å. The scattering intensity was reduced by dividing resolution and polarisation, yielding the normalized intermediate scattering functions (ISF).

Supplementary Text

Self-diffusion coefficient from pulsed-field gradient nuclear magnetic resonance

2D spectra obtained from PFG-NMR spectroscopy is fitted to the Stejskal-Tanner equation (Eq. S1) to extract the diffusion coefficient (*D*) for each proton environment. The intensity (*I*) is described by

$$I = I_0 e^{-D\gamma^2 g^2 \delta^2 \left(\Delta - \frac{\delta}{3}\right)} \quad \text{Eq. S1}$$

where I_0 is the unattenuated signal intensity, *D* is the self-correlated (Brownian) diffusion coefficient, γ is the gyromagnetic ratio of the observed nucleus (property of atom), *g* is the gradient strength, δ is the length of the gradient and *D* is the allowed diffusion time. The diffusion time and gradient length are optimised for a given sample. This allows the pulse sequence to remain constant throughout the experiment, while the gradient strength becomes the only variable parameter. The optimised measurement parameters are shown in Table S1. Measurements were repeated on different days, using slightly different parameters (e.g. longer pause between each scan or slightly longer P30). The diffusion constant obtained for each IL is within 0.1 $\times 10^{-11}$ m^2/s and the averaged value is used for ISF fitting.

Polarization analysis of NSE spectra

The normalisation of the polarisation is necessary to allow the deconvolution of coherent and incoherent fractions which carries information about different dynamic processes [1]. The coherent and incoherent scattering impact beam polarization differently. Therefore, by comparing the beam net polarization scattered from a known TiZr standard and from the sample, it is possible to deduce the relative weight of coherent and incoherent scattering events.

Upon averaging over the random nuclear spin states of the scattering system, coherent scattering occurs with no net change of neutron spin, whereas incoherent scattering has a net 2/3 probability of flipping the spin state of the scattered neutron. As a result, when the coherent:incoherent fraction of the scattering approach 1:3 (e.g., when *f* in Fig. S5 approach 0.25), the scattered beam can become depolarised, and the corresponding spin echo signal will approach 0. This yields an artefact during normalisation and incoherent correction is needed.

Activation energy for temperature dependence analysis

Limited by the number of data points, the simplest Arrhenius behaviour is assumed to estimate the effective activation energy by fitting Eq. S2.

$$D = D_0 e^{\frac{-E_a}{RT}} \quad OR \quad \tau_{Coll} = \tau_{Coll,0} e^{\frac{-E_a}{RT}} \quad \text{Eq. S2}$$

Where *D* and τ_{Coll} are the measured diffusion coefficient and fitted collective time constant from NSE respectively. E_a is the effective Arrhenius activation energy. For some ILs, particularly EAN, the weak temperature dependence precludes a physically meaningful Arrhenius description; nevertheless, the effective activation energies obtained from these fits are retained as a qualitative measure for comparing the temperature dependence of relaxation across the three ILs.

Fickian diffusion fit (single exponential model)

For hydrogen-rich samples, neutron scattering is expected to be dominated by incoherent scattering due to the large incoherent scattering cross-section of hydrogen. This mainly provides self-correlated dynamic information which should be described by

$$ISF(q,t) = K \times e^{-Dq^2t} \quad \text{Eq. S3}$$

where ISF is the normalised intermediate scattering function, K is a prefactor accounting for the normalisation artefact and D is the self-diffusion coefficient, independent of the scattering vector (q). Fitted results along with the bulk diffusion coefficient measured via PFG-NMR are shown in Fig. 2A. Fig. S1 highlights that by normalising the effective diffusion coefficient using the self-correlated diffusion coefficient obtained from NMR, all three ILs collapse onto a master curve over $q < 1.1$ Å^{-1}. This indicated the observed difference in the effective diffusion coefficient among different ILs over the low ($q < 0.6$ Å^{-1}) and intermediate ($0.6 \leq q \leq 1.1$ Å^{-1}) q-ranges scales with the bulk diffusion coefficient measured by NMR. Therefore, the sum of all dynamic processes occurring on length scales larger than the molecular dimension (> 6 Å) is strongly correlated to bulk properties. Surprisingly, this also demonstrates that the impact of IL nanostructure on transport properties is proportional across a wide range of length scales. For ion mobility overall length scales above the molecular dimension, the contribution due to the presence of polarity networks is constant, and it can be removed by normalisation using the bulk diffusion coefficient. Also, to our surprise is that this feature appears to be independent of the periodicity and correlation length of the nanostructure.

At higher q-region ($q > 1.1$ Å^{-1}), intramolecular and nearest neighbour dynamics are captured such as conformation changes and single hydrogen bonds. As a result, EtAN deviates from the master curve (Fig. S1 Right) which can be explained by the presence of its additional hydroxyl group. The contribution of intramolecular dynamics is also observed in the raw ISF (Fig. S2, S7). With increasing q, the ISF starts to show a value below 1 at $t = 0$. The residue of faster dynamics such as intramolecular dynamics can attenuate the normalised intensity at $t = 0$ [2]. This indicates the ISF starts to capture decorrelation outside the accessible time window (10^{-11} s), which is more suitably studied using techniques such as backscattering [3].

Hydrogenous datasets, Fick's diffusion + τ relaxation fits at 298 K

As discussed in the main article, the measured intermediate scattering function (*I(q,t)/I(0,t)*, ISF) from hydrogenous samples are dominated by incoherent scattering, which we interpret using a biexponential model (Eq. 1). Following an Occam's razor approach, the final model was selected as the simplest description that adequately captures the observed relaxation behaviour while minimising the number of free parameters and retaining a clear physical interpretation for each fitted parameter. Several other models were tested including stretched single-exponential model, Kohlrausch-Williams-Watts model, various forms of biexponential models, and models allowing a q-dependent relaxation of τ. Fig. S2 are data and fits for all hydrogenous ILs at 298 K. To ensure minimum number of fitted parameters are used, we first assumed the relaxation time (τ) is q-independent. Therefore, to perform a global fit, we iterated (exponential spacing) τ between 0.001 to 10 ns, with 1000 steps. At each τ, $K(q)$ and $A(q)$ were fitted at each q-value. Quality of fit is assessed by calculating the χ^2 across all ISFs. The reduced $\chi^2 > 1$ indicates that the biexponential model does not fully capture all variations in the experimental data. Nevertheless, the total χ^2 exhibits a clear minimum as a function of τ, demonstrating a well-defined best-fit

relaxation time within the accessible Fourier-time window. The uncertainty in the fitted τ, determined from the uncertainty-weighted nonlinear least-squares analysis and reported as the 1 σ standard error scaled by $\sqrt{\chi^2_{reduced}}$. It is propagated throughout the subsequent analysis. The validity of the assumption about τ being q-independent is discussed in a latter section.

K(q) and *A(q)* coefficients

The biexponential model used is provided as Eq. 1 in the main text, an example of fitted q-dependent coefficients is shown in Fig. S3. *K(q)* is a prefactor that accounts for intramolecular dynamics such as vibrations and rotations [4]. *A(q)* describes the weighting of the self-diffusion component [5]. *K(q)* is close to unity across the accessible q range and decreases only slightly at the highest q values, indicating that intramolecular motions (such as vibrations and rotations) contribute only marginally and can be neglected. In contrast, over the same range of length scales, *A(q)* decreases from 1 to 0 with increasing q, reflecting the growing contribution of the fast relaxation mode at shorter length scales.

q-dependence of the τ relaxation time

The τ relaxation time is assumed to be a constant across the entire accessible q-range in Eq.1. To test this is valid, we have tried to fit it as a q-dependent variable. As an example, the fit and the fitted τ for fully hydrogenous EAN are shown in Fig. S4. While the q-dependence slightly improved the quality of fit, it is clear that τ is a constant across the q-range where these fast sub-diffusive modes contribute significantly to the total dynamics ($q > 0.6$ Å^{-1}). That is, the obtained ISF alone does not include sufficient information to disentangle the q-dependence of the fast sub-diffusive mode.

Selective deuteration

To adjust the relative weighting of coherent and incoherent contributions, we have selectively substituted hydrogen (H) with deuterium (D). Deuterium has a much smaller incoherent scattering cross section. ILs with more deuterium display higher coherent fractions (Fig. S5). The three versions of ILs are outlined below in Table S2. Despite the two deuterated contrasts having different scattering cross sections, we have treated the underlying dynamics to be identical since all deuterium are on the cation.

Incoherent correction

As outlined in the main article we have performed incoherent corrections to deuterated datasets to isolate the coherent dynamics. The correction is performed by

$$r_{Inc} = \frac{f_{Inc}}{f_{Coh}} = \frac{1 - f_{Coh}}{f_{Coh}}$$
$$ISF(q,t)_{Corr} = ISF(q,t) \times \left(1 - \frac{r_{Inc}}{3}\right) + ISF(q,t)_{Inc} \times \frac{r_{Inc}}{3} \qquad \text{Eq. S4}$$

where f_{Coh} is the experimental coherent fraction (Fig. S4); $ISF(q,t)_{Corr}$, $ISF(q,t)$ and $ISF(q,t)_{Inc}$ are corrected, experimental (for deuterated datasets), and incoherent intermediate scattering functions. The incoherent ISF is obtained from hydrogenous dataset based on Eq. 1, the best fit values of *K(q)*, *A(q)* and τ_{Self} were used to generate the incoherent ISF. This should remove most incoherent contributions from the deuterated datasets and further amplify the coherent contribution within the corrected ISFs.

There are two distinct sources of incoherent scattering, spin and isotope. Spin incoherence occurs when nuclei have randomly oriented spin states, leading to fluctuations in the scattering interaction because the scattering length depends on the relative spin configuration. Since these spin states are typically uncorrelated, the scattered waves do not interfere constructively, producing an incoherent background signal. Isotope incoherence, on the other hand, results from the presence of different isotopes of the same element, which have different nuclear scattering lengths. The random distribution of these isotopes in a sample causes variations in scattering strength from site to site, again preventing coherent interference.

The incoherent correction accounts for the spin incoherence and uses the experimental coherent fraction, it lacks description of any isotope incoherence since hydrogenous dataset will contain negligible deuterium. However, in partially and fully deuterated datasets, at least 1% of all hydrogen sites will be a different isotope to the dominate hydrogen isotope. As a result, the incoherent correction described above cannot remove all incoherent contributions. Therefore, the following expression analogous to Eq. 1 is adopted to interpret the partially and fully deuterated datasets.

$$I(q,t)/I(q,0)_{Coh} = K(q) \times \mathrm{e}^{-D_{NMR}q^2t} \times \left(A(q) + \left(1 - A(q)\right) \times e^{\frac{-t}{\tau_{Coll}}}\right) \qquad \text{Eq. S5}$$

All variables are defined identically to Eq. 1 apart from τ_{Coll}, which now captures a coherent mode that describes collective dynamics. While incoherent contributions are removed to the extent possible, we consistently observe a slow relaxation that follows q^{-2} dependence. This is characteristic to self-diffusion; hence we have attributed it to residue incoherent contributions. Although the magnitude of this contribution is substantially lower than within the hydrogenous dataset, due to its relatively slow nature, it is recognizable especially at low q. Therefore, in Eq. S5 we have included the same Fickian term and fixed the self-diffusion coefficient to experimental values obtained via PFG-NMR. A representative incoherent correction is shown in Fig. S6, all fits to the deuterated datasets are shown in Fig. S7. Fitted τ_{Coll} are shown in Table S3.

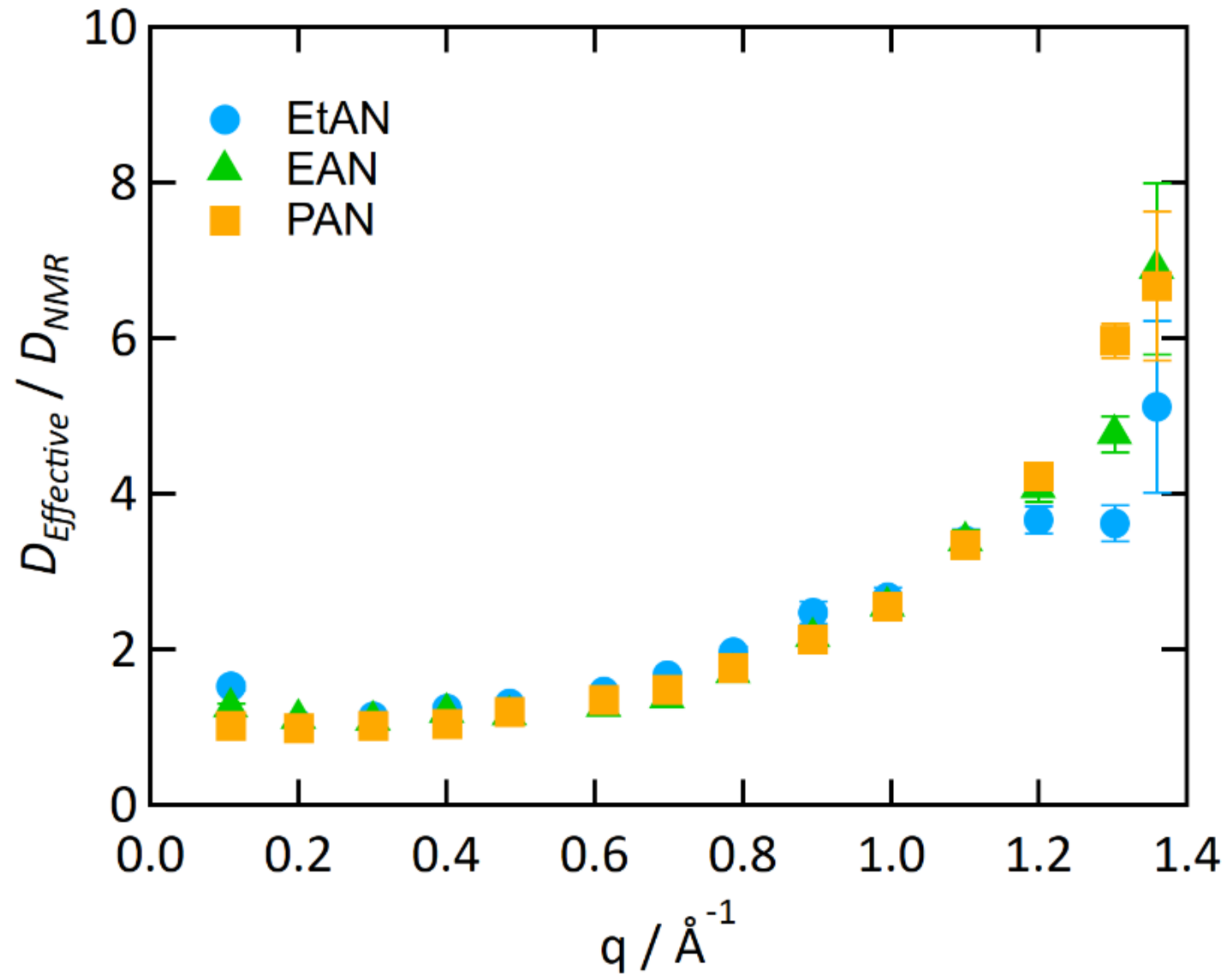

Fig. S1. Unitless dynamics at different scattering vectors. The effective diffusion constant was calculated by fitting the hydrogenous ISF to a single exponential decay (Eq. S3), then divided by the self-diffusion coefficient measured via NMR.

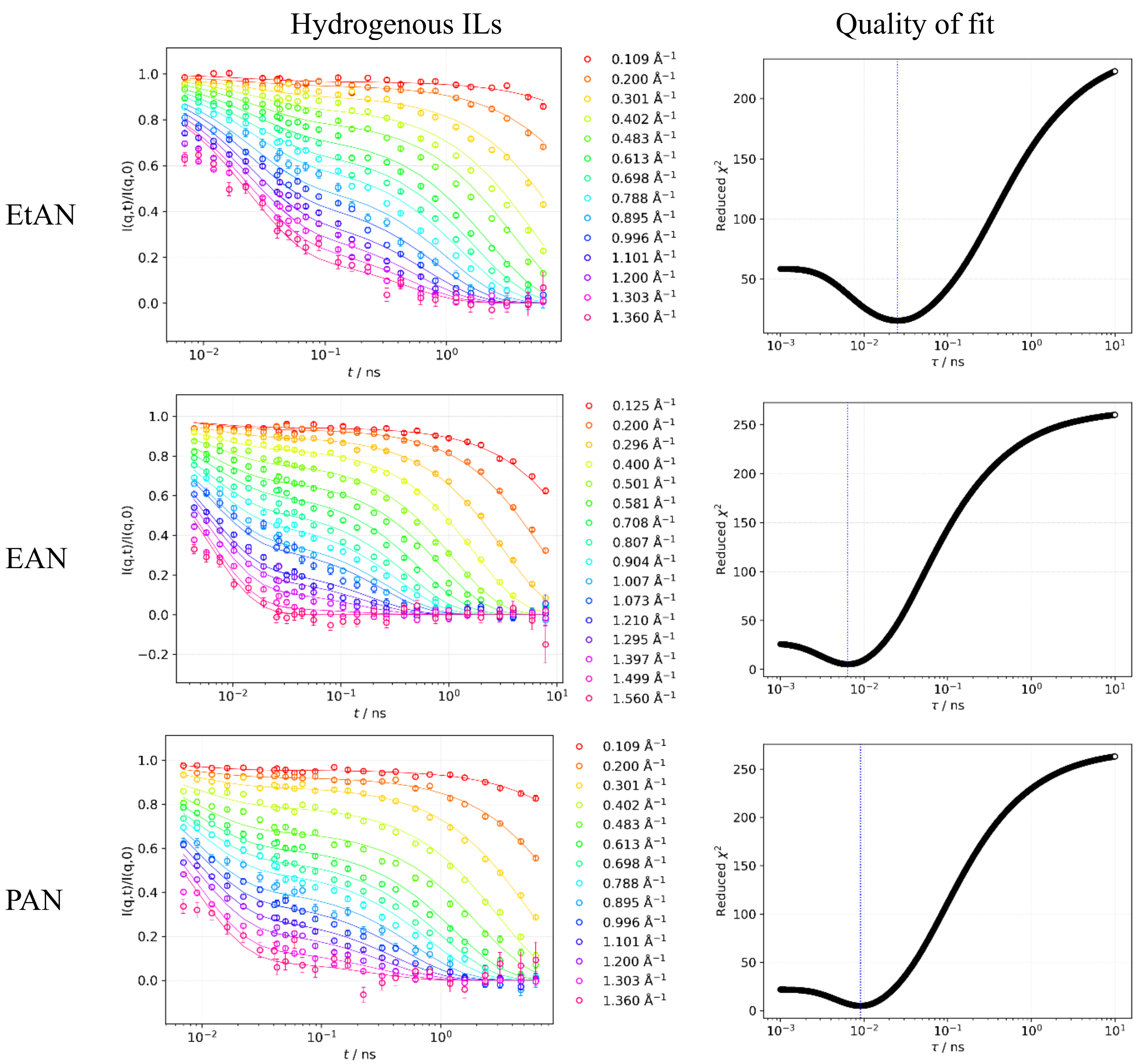

Fig. S2. ISFs obtained from WASP at 298 K with hydrogenous ILs. Hollow markers are experimental results, and solid lines are global fits according to Eq. 1. From top to bottom are for EtAN, EAN and PAN. The right panel plots the χ^2 vs τ, which confirms a unique best-fit τ relaxation time exist for all datasets within the accessible Fourier time range.

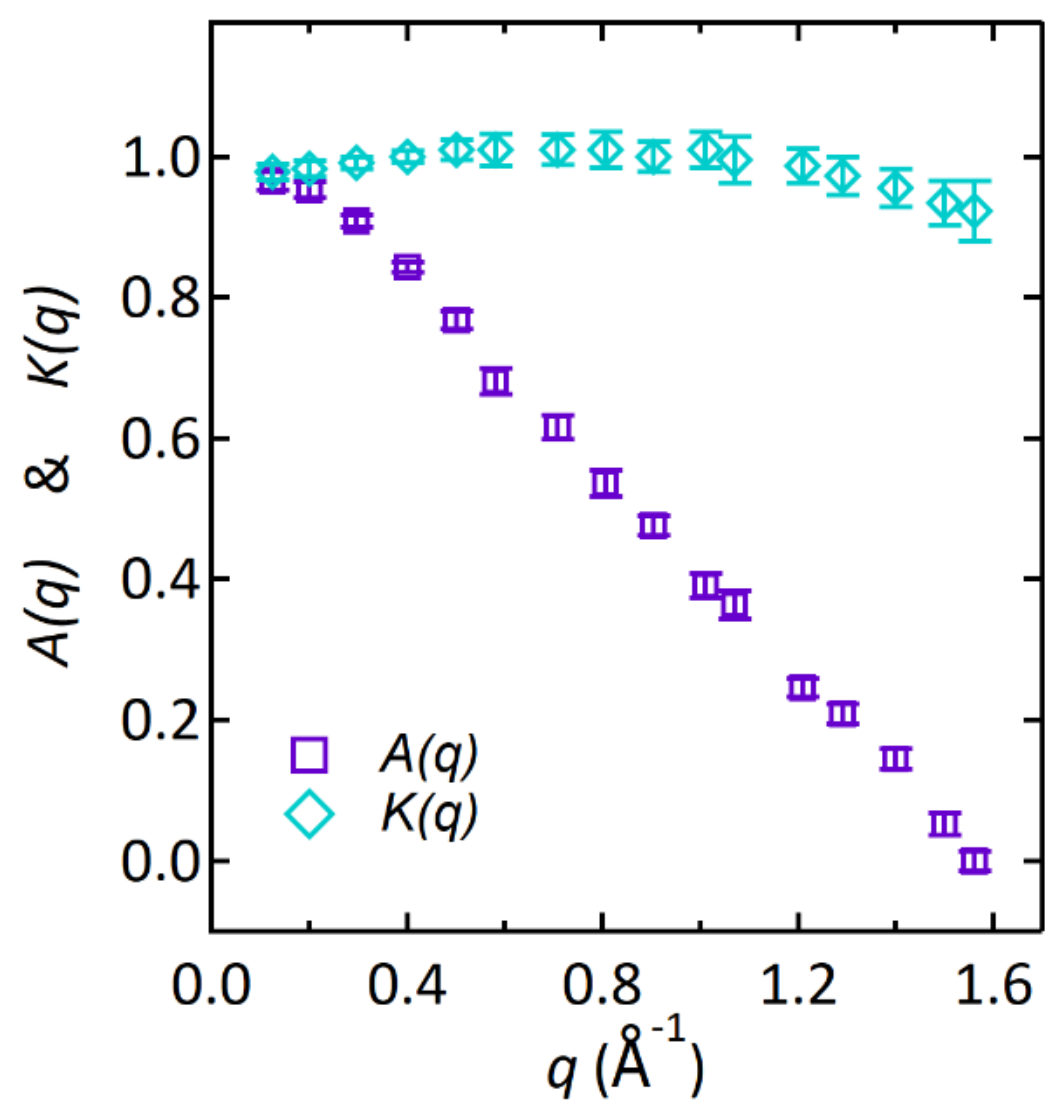

Fig. S3. Fitted *K(q)* and *A(q)* coefficients for hydrogenous EAN, shown in Fig. 2B and replotted in Fig S2.

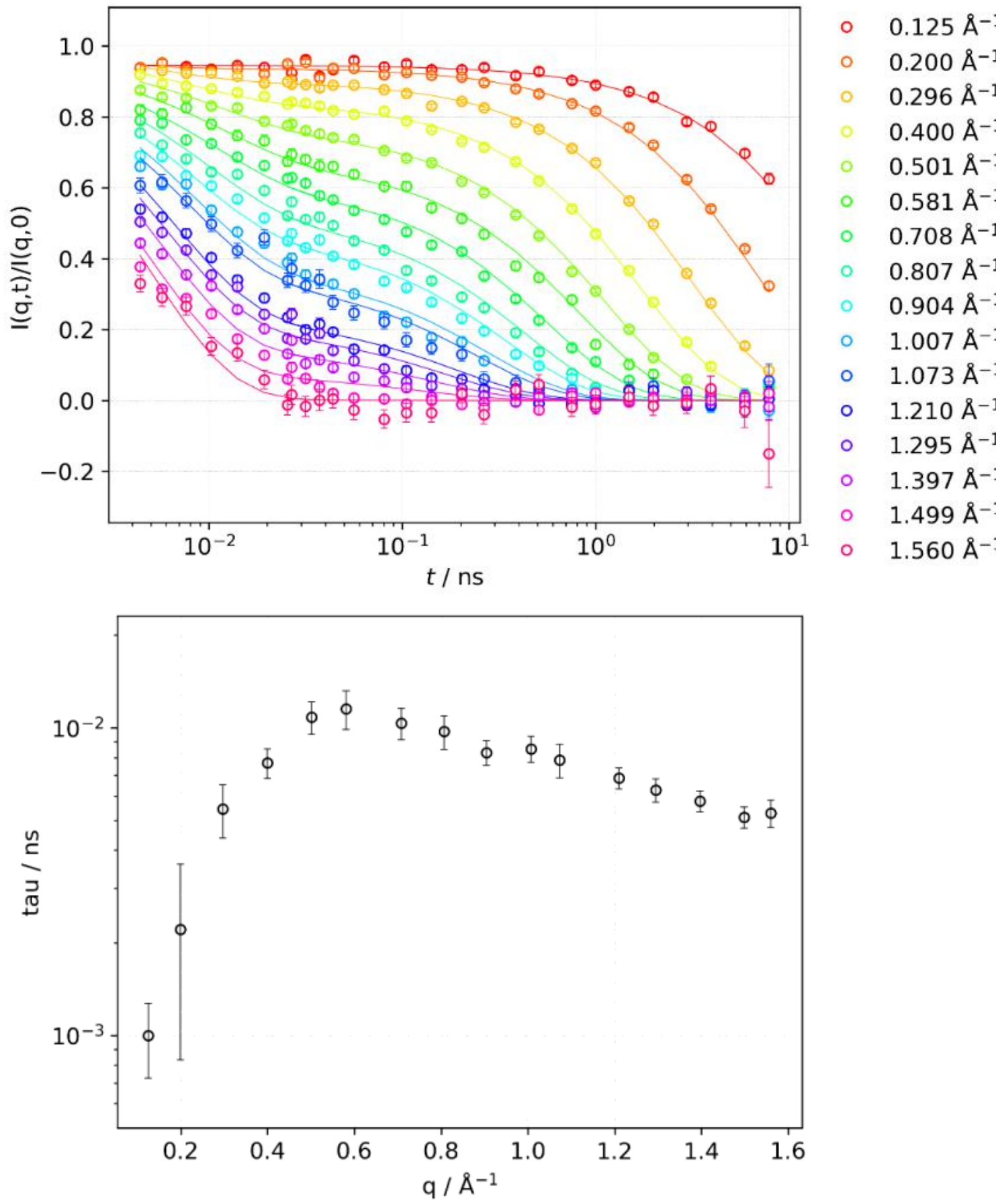

Fig. S4. TOP: ISFs obtained from at 298 K with hydrogenous EAN Hollow markers are experimental results and solid line are fits. BOTTOM: Best fit q-dependent t relaxation time for fully hydrogenous EAN at 298. It shows that τ is effectively a constant for $q > 0.6$ Å^{-1}.

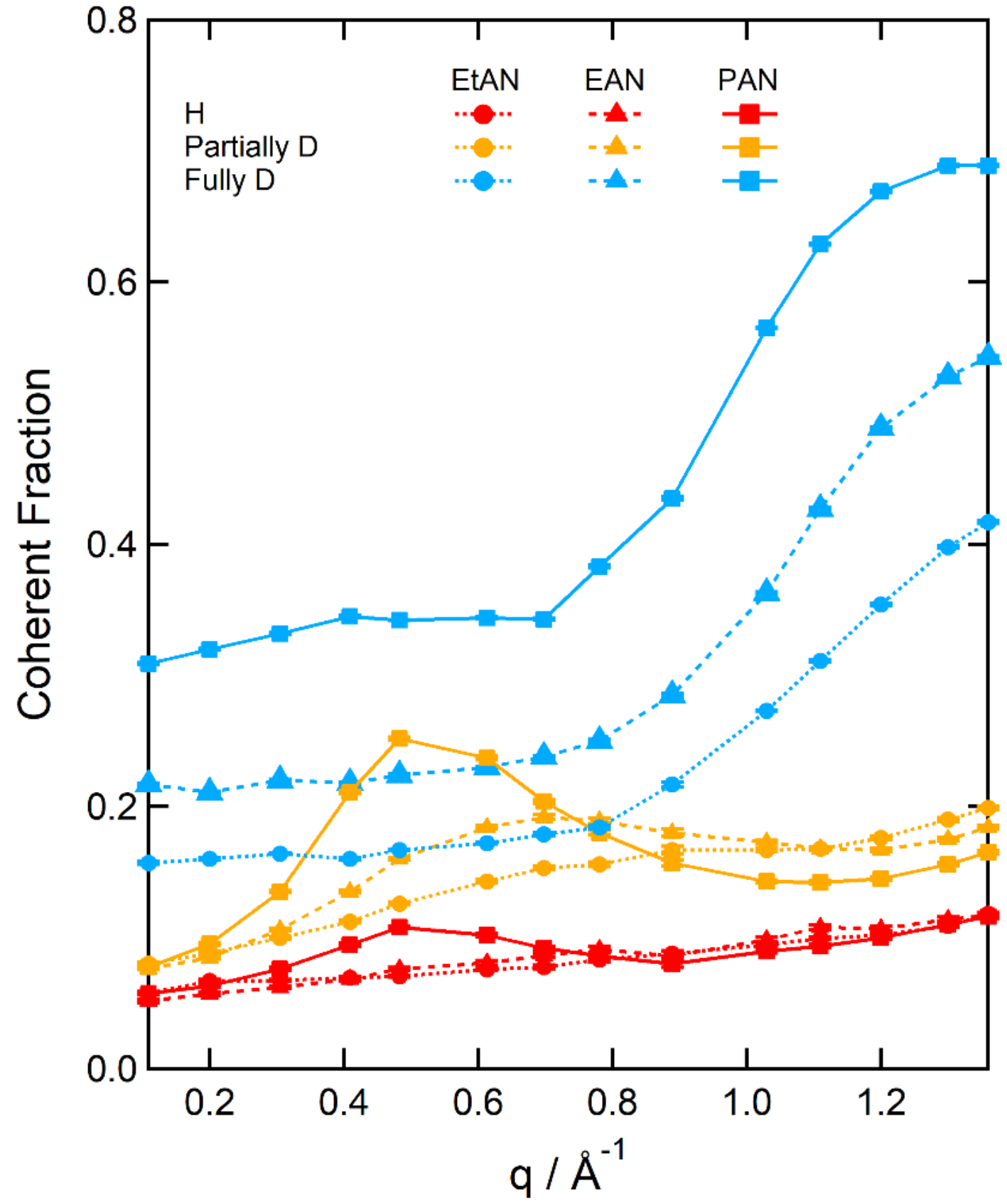

Fig. S5. Fraction of elastically scattered neutron as functions of scattering vector, *q*. Determined by polarization analysis [1, 6]. Lines between markers are added to guide the eye. For all ILs, the coherent fraction follows fully deuterated > partially deuterated > hydrogenous. A clear peak in coherent scattering is present in EAN and PAN, consistent with their known nanostructure. The experimentally measured coherent scattering fraction for all hydrogenous ILs is always below 0.12 across the entire accessible *q* range. PAN shows a marked peak close to its nanostructure peak (Figure 1), EAN and EtAN to a lesser extent, consistent with static scattering results [7, 8].

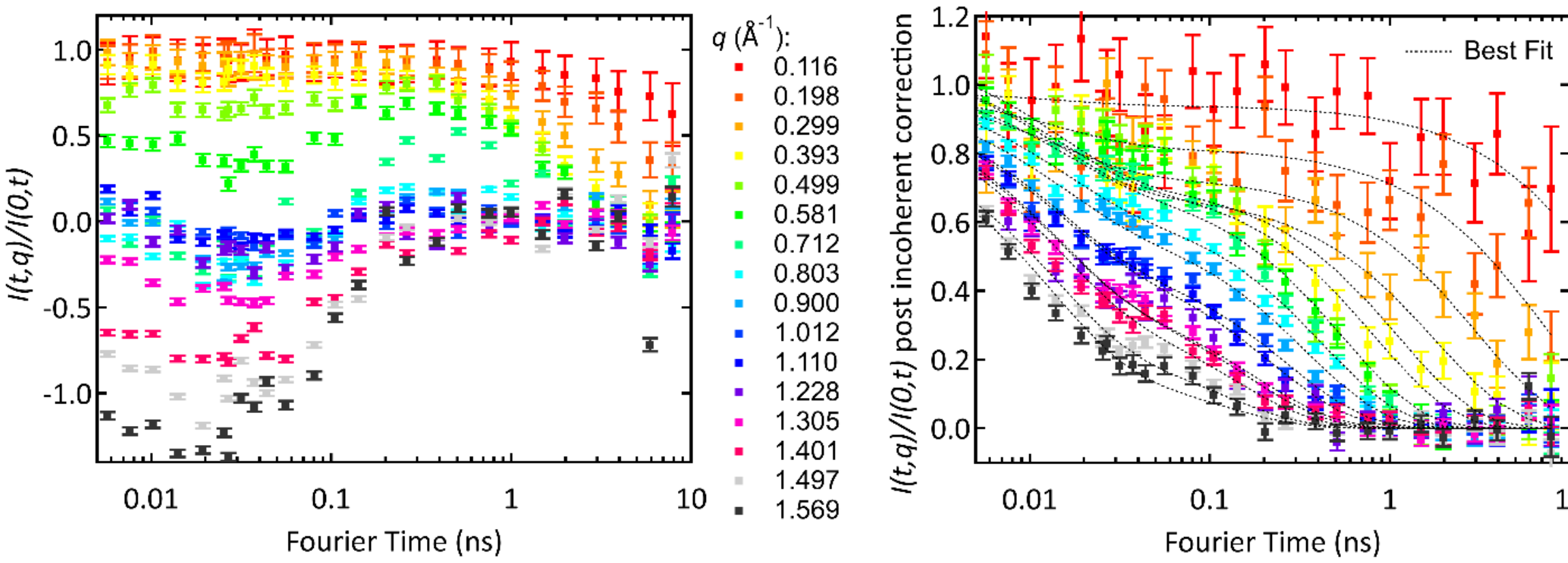

Fig. S6. Raw (Left) and incoherent corrected (Right) ISF for partially deuterated EAN at 298 K.

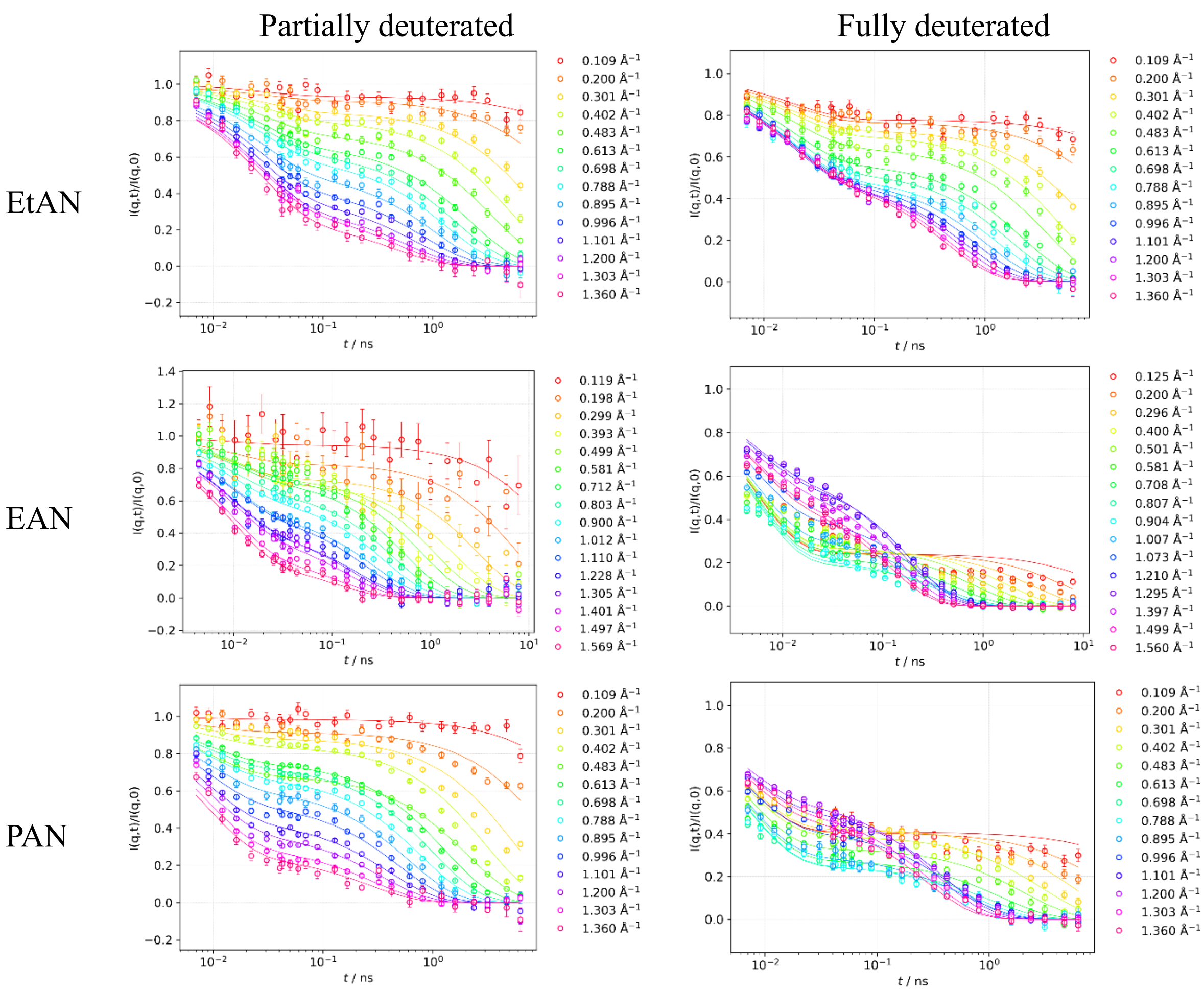

Fig. S7. ISFs obtained from WASP at 298 K with deuterated ILs. Hollow markers are experimental results and solid line are global fits according to Eq. S4. Results are summarised in Table S3.

Table S1. Optimised parameters for performing PFG_NMR measurement of neat alkylammonium ILs at 298 K.

IL	P30 (ms), relates to *d*	D20 (ms), relates to *D*
EtAN	3.000	200
EAN	2.500	150
PAN	3.000	150

Table S2. Condensed structural formula of ethanolammonium nitrate (EtAN), ethylammonium nitrate (EAN) and propylammonium nitrate (PAN) with partial and full deuterium substitution. Deuterium is highlighted in bold.

IL	Hydrogenous	Partially Deuterated	Fully Deuterated
EtAN	$[HOCH_2CH_2NH_3]^+ [NO_3]^-$	$[\mathbf{D}OCH_2CH_2N\mathbf{D_3}]^+ [NO_3]^-$	$[\mathbf{D}OC\mathbf{D_2}C\mathbf{D_2}N\mathbf{D_3}]^+ [NO_3]^-$
EAN	$[CH_3CH_2NH_3]^+ [NO_3]^-$	$[CH_3CH_2N\mathbf{D_3}]^+ [NO_3]^-$	$[C\mathbf{D_3}C\mathbf{D_2}N\mathbf{D_3}]^+ [NO_3]^-$
PAN	$[CH_3CH_2CH_2NH_3]^+ [NO_3]^-$	$[CH_3CH_2CH_2N\mathbf{D_3}]^+ [NO_3]^-$	$[C\mathbf{D_3}C\mathbf{D_2}C\mathbf{D_2}N\mathbf{D_3}]^+ [NO_3]^-$

Table S3. Fitted incoherent τ_{Self} relaxation time from hydrogenous ILs and fitted coherent (collective) relaxation time from partially ($\tau_{Coll, part}$) and fully deuterated ILs ($\tau_{Coll, full}$), all at 298 K. Viscosities are taken from literature [9].

IL	τ_{Self} (10^{-12} s)	$\tau_{Coll, part}$ (10^{-12} s)	$\tau_{Coll, full}$ (10^{-12} s)	Viscosity (mPa s)
EtAN	25.3 ± 1.5	28.8 ± 0.7	18.4 ± 0.4	128
EAN	6.4 ± 0.2	10.2 ± 0.4	5.5 ± 0.2	32.1
PAN	9.0 ± 0.2	9.1 ± 0.1	6.9 ± 0.2	73.7

Table S4. Temperature dependence of experimental self-diffusion coefficient (D_{NMR}), τ_{Self}, and τ_{Coll}, as well as the calculated Arrhenius activation energy (E_a).

	EtAN	EAN	PAN
$D_{NMR,\ 298\ K}$ (10^{-11} m^2/s)	1.20 ± 0.08	3.58 ± 0.05	2.04 ± 0.01
$D_{NMR,\ 318\ K}$ (10^{-11} m^2/s)	3.05 ± 0.18	6.80 ± 0.13	4.21 ± 0.02
$D_{NMR,\ 338\ K}$ (10^{-11} m^2/s)	5.97 ± 0.25	11.5 ± 0.06	7.54 ± 0.03
$E_{a,\ NMR}$ (kJ /mol)	33.7 ± 1.9	24.5 ± 0.5	27.4 ± 0.7
$\tau_{Self,\ 298\ K}$ (10^{-12} s)	25.3 ± 1.5	6.4 ± 0.2	9.0 ± 0.2
$\tau_{Self,\ 318\ K}$ (10^{-12} s)	15.8 ± 0.6	6.8 ± 0.3	8.6 ± 0.2
$\tau_{Self,\ 338\ K}$ (10^{-12} s)	14.8 ± 0.6	6.4 ± 0.2	6.4 ± 0.2
$E_{a,\ Self}$ (kJ /mol)	11.3 ± 2.7	----	6.3 ± 0.8
$\tau_{Coll,\ 298\ K}$ (10^{-12} s)	18.4 ± 0.4	5.5 ± 0.2	6.9 ± 0.2
$\tau_{Coll,\ 318\ K}$ (10^{-12} s)	11.5 ± 0.2	6.4 ± 0.1	6.5 ± 0.2
$\tau_{Coll,\ 338\ K}$ (10^{-12} s)	10.8 ± 0.2	5.6 ± 0.1	5.6 ± 0.1
$E_{a,\ Coll}$ (kJ /mol)	11.3 ± 4.5	----	4.3 ± 1.2

References (all cited in the main text):

(1) Arbe, A.; Nilsen, G. J.; Devonport, M.; Farago, B.; Alvarez, F.; Martínez González, J. A.; Colmenero, J. Collective dynamics and self-motions in the van der Waals liquid tetrahydrofuran from meso- to inter-molecular scales disentangled by neutron spectroscopy with polarization analysis. *The Journal of Chemical Physics* **2023**, *158* (18), 184502. DOI: 10.1063/5.0147427.

(2) Ferdeghini, F.; Berrod, Q.; Zanotti, J. M.; Judeinstein, P.; Sakai, V. G.; Czakkel, O.; Fouquet, P.; Constantin, D. Nanostructuration of ionic liquids: impact on the cation mobility. A multi-scale study. *Nanoscale* **2017**, *9* (5), 1901-1908, Article. DOI: 10.1039/c6nr07604a.

(3) Gardner, J. S.; Ehlers, G.; Faraone, A.; García Sakai, V. High-resolution neutron spectroscopy using backscattering and neutron spin-echo spectrometers in soft and hard condensed matter. *Nature Reviews Physics* **2020**, *2* (2), 103-116. DOI: 10.1038/s42254-019-0128-1.

(4) Russina, M.; Günther, G.; Farago, B.; Babcock, E.; Salhi, Z.; Ioffe, A.; Mezei, F. Structural Fluctuations at Nanoscale and Cooperative Molecular Dynamics in Bulk Water. *The Journal of Physical Chemistry Letters* **2025**, *16* (23), 5835-5843. DOI: 10.1021/acs.jpclett.5c00735.

(5) Arbe, A.; Arrese-Igor, S.; Ruiz-Martín, M. D.; Farago, B.; Alvarez, F.; Nilsen, G. J.; Colmenero, J. Mesoscopic Dynamics in Molecular Liquids: Universality of Nondispersive Structural Mode and Its Reflection in Self-Atomic Motions. *Phys. Rev. Lett.* **2025**, *134* (9), 098001. DOI: 10.1103/PhysRevLett.134.098001.

(6) Kofu, M.; Nagao, M.; Ueki, T.; Kitazawa, Y.; Nakamura, Y.; Sawamura, S.; Watanabe, M.; Yamamuro, O. Heterogeneous Slow Dynamics of Imidazolium-Based Ionic Liquids Studied by Neutron Spin Echo. *The Journal of Physical Chemistry B* **2013**, *117* (9), 2773-2781. DOI: 10.1021/jp312608r.

(7) Hayes, R.; Imberti, S.; Warr, G. G.; Atkin, R. Effect of Cation Alkyl Chain Length and Anion Type on Protic Ionic Liquid Nanostructure. *The Journal of Physical Chemistry C* **2014**, *118*, 13998-14008. DOI: 10.1021/jp503429k.

(8) Hayes, R.; Imberti, S.; Warr, G. G.; Atkin, R. Amphiphilicity determines nanostructure in protic ionic liquids. *Physical Chemistry Chemical Physics* **2011**, *13* (8), 3237-3247. DOI: 10.1039/C0CP01137A.

(9) Smith, J. A.; Webber, G. B.; Warr, G. G.; Atkin, R. Rheology of Protic Ionic Liquids and Their Mixtures. *The Journal of Physical Chemistry B* **2013**, *117* (44), 13930-13935. DOI: 10.1021/jp407715e.
